# Granzyme B Attenuates Bacterial Virulence by Targeting Secreted Factors

**DOI:** 10.1016/j.isci.2020.100932

**Published:** 2020-02-21

**Authors:** Diego López León, Patricia Matthey, Isabelle Fellay, Marianne Blanchard, Denis Martinvalet, Pierre-Yves Mantel, Luis Filgueira, Michael Walch

**Affiliations:** 1 Faculty of Science and Medicine, Department of Oncology, Microbiology and Immunology, Anatomy Unit, University of Fribourg, PER03.14, Route Albert Gockel 1, 1700 Fribourg, Switzerland; 2 Department of Biomedical Sciences, University of Padova, Via Ugo Bassi 58/B, 35121 Padova, Italy

**Keywords:** Biological Sciences, Immunology, Immune Response, Microbiology

## Abstract

Pathogenic bacteria secrete virulence factors that interact with the human host to establish infections. The human immune system evolved multiple mechanisms to fight bacterial invaders, including immune proteases that were demonstrated to contribute crucially to antibacterial defense. Here we show that granzyme B degrades multiple secreted virulence mediators from *Listeria monocytogenes*, *Salmonella typhimurium*, and *Mycobacteria tuberculosis*. Pathogenic bacteria, when infected in the presence of granzyme B or granzyme-secreting killer cells, fail to grow in human macrophages and epithelial cells owing to their crippled virulence. A granzyme B-uncleavable mutant form of the major *Listeria* virulence factor, listeriolysin O, rescued the virulence defect in response to granzyme treatment. Hence, we link the degradation of a single factor with the observed decrease in virulent bacteria growth. Overall, we reveal here an innate immune barrier function of granzyme B by disrupting bacterial virulence to facilitate bacteria clearance by bystander immune and non-immune cells.

## Highlights

• Granzyme B specifically degrades secreted virulence factors of three bacterial species
• Various killer cells secrete granzyme B upon contact with gram− and gram+ bacteria
• Granzyme B is co-endocytosed into host cells with bacteria to inhibit their growth
• Uncleavable listeriolysin O rescues *Listeria* virulence in response to granzyme B

## Graphical Abstract

**Figure:**
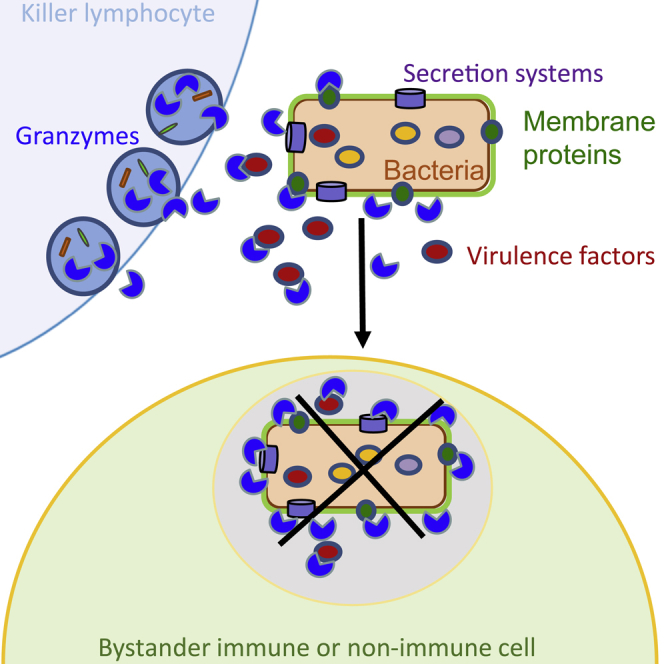

## Introduction

Pathogenic, facultatively intracellular bacteria, such as *Listeria*, *Salmonella*, and *Mycobacteria*, are a global major health threat because they cause infections in human hosts leading to severe disease and death if untreated. Treatment nowadays is often complicated because of the massive increase in antimicrobial resistance (Prestinaci et al., 2015). To survive in the host, pathogenic bacteria evolved multiple secreted or externally exposed virulence mediators that allow specific interaction with the host cell. These factors orchestrate the major steps in a bacterial virulence strategy, which help with adherence, cellular invasion, and, ultimately, the setup of a protective niche. The virulence factors enable interference with multiple biological pathways of the host, including host defense. Virulence allows enhanced bacterial multiplication or persistence in the host that causes tissue damage and disease. Finally, successful virulent bacteria manage to exit and transmit the infection to a new host (Webb and Kahler, 2008). The virulence strategy of *Listeria monocytogenes* responsible is characterized by various mechanisms, including forced uptake even in non-phagocytic cells, such as epithelial cells. After uptake, they avoid lethal lysosomal degradation by a phagosomal escape mechanism, which is mainly promoted by their major virulence factor, listeriolysin O (LLO), supported by a few phospholipases. In the cytosol, *Listeria* rapidly multiply and specifically interact with the actin cytoskeleton to gain motility for cell-to-cell spreads (Cossart, 2011).

On the other hand, the immune system evolved multiple strategies to fight bacterial pathogens. Innate immune mechanisms act in a coordinated fashion to restrict an infection. Among those, innate cytotoxic lymphocytes, in particular, natural killer and γδ T cells are critical in the early defense against invading pathogens via the production of inflammatory cytokines and direct bactericidal activities (Gao and Williams, 2015, Gregory et al., 1996, Zheng et al., 2013). Immune proteases, such as the neutrophil serine proteases, have been recognized to be essential for innate antibacterial immune defense (Stapels et al., 2015). Mice deficient in cathepsin G and neutrophil elastase are greatly impaired in the elimination of gram-positive and gram-negative bacteria (Hahn et al., 2011, Hirche et al., 2008, Steinwede et al., 2012).

We recently discovered that GzmB efficiently kills bacteria when delivered into the bacterial cytosol by the membrane disrupting protein, granulysin (GNLY) (Walch et al., 2014). Although the concerted action of these two cytotoxic molecules seems an attractive model for the elimination of intracellular bacteria, multiple questions, particularly concerning the GNLY delivery mechanism, remain problematic. First, GNLY is not expressed in rodents (Krensky and Clayberger, 2009) despite their potent immunity against bacterial invaders, such as *Listeria monocytogenes* (Pamer, 2004). Second, GNLY oddly displays virtually no membranolytic activity at the physiological NaCl concentration of 140 mM and neutral pH, questioning any contribution to antibacterial immune response in the extracellular space (Barman et al., 2006, Ernst et al., 2000). Third, GNLY expression is exclusively limited to activated lymphocytes (Krensky and Clayberger, 2009). Therefore, GNLY will be predominantly released into immunological synapses between lymphocytes and bacterial-infected target cells. On the other hand, multiple cell types secrete Gzms, in particular GzmB, in response to inflammatory stimuli. This growing list includes myeloid cells, such as monocytes (Elavazhagan et al., 2015), mast cells (Strik et al., 2007), and neutrophils (Mattila et al., 2015, Wagner et al., 2008), as well as plasmacytoid dendritic cells (Rissoan et al., 2002) and B cells (Hagn and Jahrsdorfer, 2012, Lindner et al., 2013), and even non-immune cells, such as smooth muscle cells, keratinocytes, and chondrocytes (Boivin et al., 2009). Furthermore, extracellular granzymes are elevated in patients with bacterial infections (Lauw et al., 2000). We therefore asked if there is a GNLY-independent antimicrobial activity of GzmB.

## Results

### GzmB Cleaves Multiple Secreted or Membrane-Exposed Bacterial Virulence Factors

**Figure 1:**
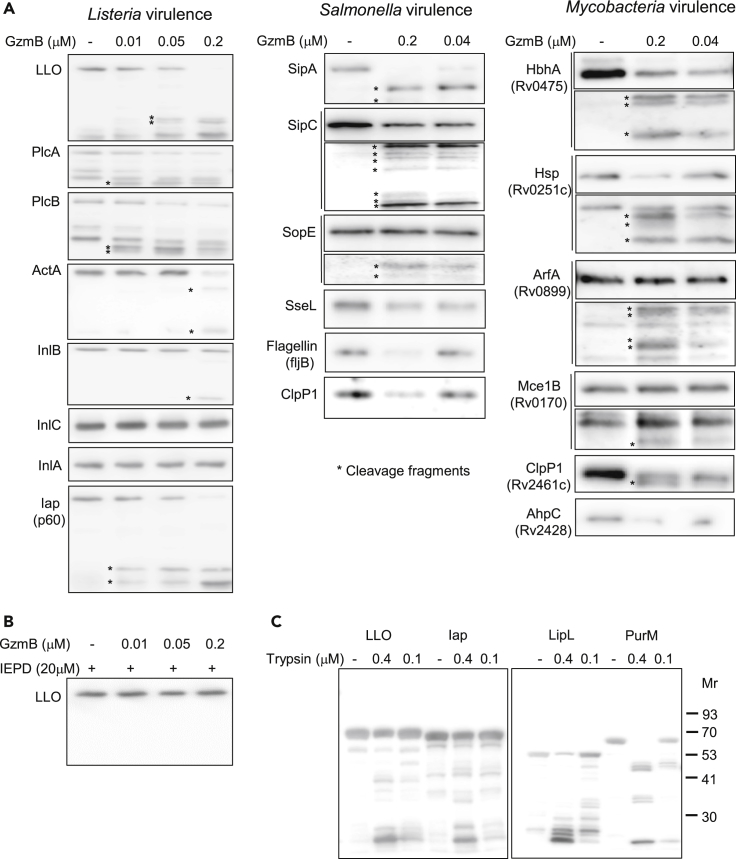
GzmB Cleaves Secreted or Membrane-Exposed Bacterial Virulence Factors (A–C) (A) *E. coli* ectopically expressing GST-tagged *Listeria monocytogenes*, *Salmonella typhimurium*, or *Mycobacteria tuberculosis* proteins were treated with lysozyme and hypotonically lysed by freeze-thaw. Crude lysates were treated with indicated concentrations of GzmB for 20 min (A and B) or with the unspecific serine protease, trypsin (C), for 5 min before substrate cleavage was assessed by anti-GST immunoblots. To highlight both the decrease of the full-length protein and the appearance of cleavage fragments, some blots (SipC, SopE, HBhA, Hsp, ArfA, and Mce1B) were divided and presented at different exposure times. The full lanes at medium exposure times are shown in Figure S2. In (B), the GzmB treatment of LLO was performed in the presence of the GzmB inhibitor Ac-IEPD-CHO.

Without cytosolic delivery by GNLY, the Gzms can only target secreted or externally exposed bacterial proteins, known to be involved in virulence (Desvaux and Hebraud, 2006, Sharma et al., 2017). When we treated *Listeria monocytogenes* (*Lm*), strain10403S, supernatants with GzmB, we found that two major virulence factors, listeriolysin O (LLO) and the invasion associated protein p60 (Iap), were efficiently degraded by the protease (Figure S1A). Using an ectopic expression system and immunoblotting, we systemically tested the canonical, positive regulator factor A (PrfA)-dependent *Lm* virulence mediators (Scortti et al., 2007) in addition to Iap for GzmB susceptibility. GzmB cleaved most of these virulence factors. The exemptions were internalin C (InlC, very low efficiency, cleavage band only after longer GzmB exposure, Figure S2) and InlA (Figure 1). The degradation depended on GzmB activity as the presence of the specific GzmB inhibitor completely abrogated the cleavage of the highly susceptible substrate LLO (Figure 1B). Strikingly, GzmB most efficiently degraded the membranolytic factors, LLO, and the two major phospholipases, PlcA and PlcB. This suggested that two crucial steps in the *Lm* virulence strategy, the phagosomal escape and cell-to-cell spread (Seveau et al., 2007), might be disrupted in response to GzmB. Five randomly picked cytosolic listerial proteins were not affected by the protease (Figure S1B), suggesting specific targeting of secreted *Lm* virulence factors by GzmB. The unspecific serine protease, trypsin, degraded the *Listeria* virulence factors, LLO and Iap, as well as the cytosolic proteins, LipL and PurM, with high efficiency (Figure 1C) and even GST alone that was also not affected by GzmB (Figure S1C).

To exclude that the targeting of bacterial virulence mediators by the immune protease is a phenomenon specific to *Lm*, we assessed the impact of GzmB on various well-characterized secreted or externally exposed virulence mediators of *Salmonella typhimurium*, strain SL1344, and *Mycobacteria tuberculosis*, strain H37Rv. Surprisingly, GzmB degraded all of the tested bacterial factors (Figure 1A), although some of them with rather low efficiency. The full lanes of the cleavage blots are demonstrated in Figure S2. However, bacterial factors essential for adhesion and invasion, such as the *Salmonella* invasion proteins SipA and SipC (Ibarra and Steele-Mortimer, 2009) or the mycobacterial Heparin-binding hemagglutinin (HbhA) (Forrellad et al., 2013), were cleaved very efficiently, pointing again toward a defect early during infection in response to GzmB. As for *Lm*, randomly picked cytosolic control proteins of the other two bacterial strains were not degraded by the protease (Figure S1B) suggesting a specific attack on external bacterial virulence factors by GzmB.

### GzmB Inhibits LLO Hemolytic Activity and *Listeria* Phagosomal Escape in Human Macrophages upon Co-endocytosis with Bacteria into Host Cells

**Figure 2:**
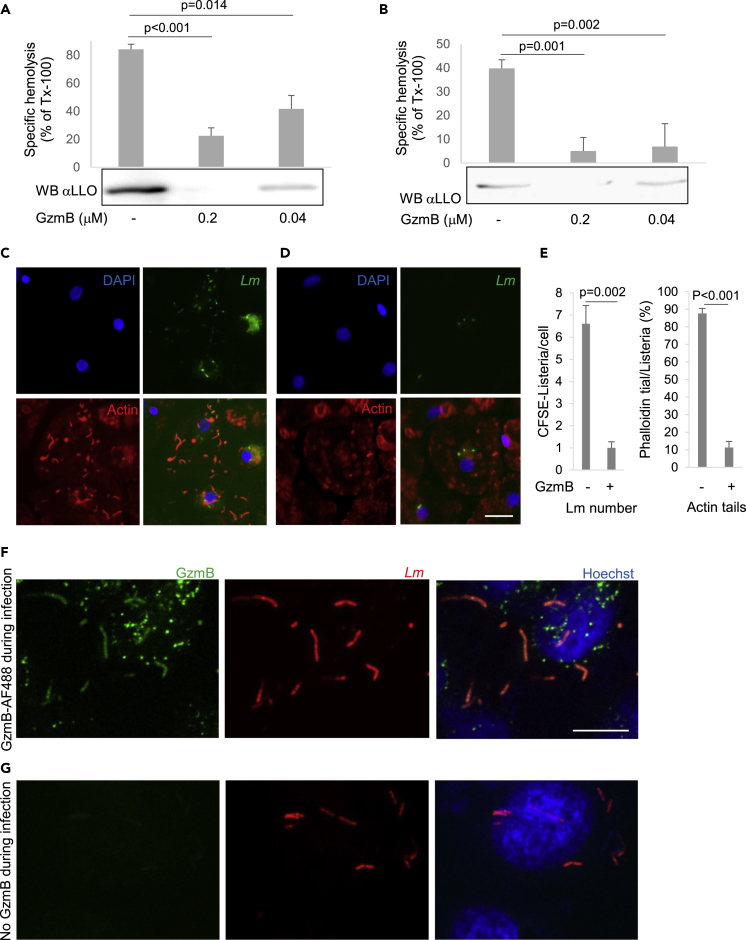
GzmB Disrupts LLO Hemolytic Activity and *Listeria* Phagosomal Escape in Human Macrophages upon Co-endocytosis with Bacteria into the Host Cells Recombinant LLO (A) or *Listeria monocytogenes-*secreted proteins in supernatants (B) were treated with indicated concentrations of GzmB for 20 min before the protease was inactivated with DCI for 30 min on ice. These samples were used to treat human red blood cells for 15 min at 37°C. Hemoglobin release was measured spectrophotometrically at 405 nm wavelength by plate reader and is presented as average ± SEM of three independent experiments. Cleavage of LLO assessed by immunoblot using an anti-LLO antibody. An overexposed image of the blot illustrating the cleavage fragments of native LLO in the supernatant after GzmB treatment is shown in Figure S3. Human macrophages were infected with CFSE-labeled *Listeria monocytogenes* in the absence (C) or presence of 0.2 μM GzmB (D) before being fixed and stained with phalloidin-AF594 (red) and DAPI (blue). In (E), *Listeria* were counted and analyzed in 10 visual fields (n > 100) per condition in three independent experiments. HeLa cells were infected with PHK26 red-labeled *Listeria monocytogenes* in the presence of 0.2 μM GzmB-AF488 (F) or left untreated (G) before being fixed and stained with Hoechst (blue) for high resolution confocal microscopy analysis, bars are 10 μm. Representative maximum projections of z stack images (eight stacks, step size 0.5 μm) from independently repeated experiments are shown.

The functional impact on LLO by GzmB-mediated degradation was assessed in hemolysis assays. For this purpose, purified recombinant LLO (Figure 2A), as well as *Lm* supernatants containing secreted virulence factors (Figure 2B), was pretreated with GzmB before incubation with red blood cells to assess hemolytic activity. Indeed, GzmB mediated LLO degradation, verified by the decreased LLO signal intensity in immunoblots (and the appearance of typical cleavage fragments in Figure S3), and significantly inhibited the hemolytic activity, therefore disrupting the enzymatic function of this virulence mediator. LLO, supported by PlcA and PlcB, is essential for *Lm* phagosomal escape (Hamon et al., 2012). After the escape, *Lm* interacts with actin to form comet-shaped tails that can be visualized and quantified by fluorescence microscopy using phalloidin staining. When we infected human macrophages under control conditions, most of the numerous intracellular bacteria displayed an actin tail 5 h post infection (Figures 2C and 2E). However, when we infected the cells in the presence of GzmB, both the *Listeria* number and the actin recruitment of the intracellular bacteria were significantly reduced (Figures 2D and 2E). In addition, we found that, at the initial infection stage (1 h), the bacteria are covered by fluorescently labeled GzmB indicating co-endocytosis of the enzymes together with bacteria into host cells (Figures 2F and 2G). This may lead to increased GzmB concentration in the bacteria-containing compartment during the initial infection and therefore facilitating virulence factor degradation.

### GzmB Treatment during Infection Inhibits Growth of Intracellular *Listeria* and *Salmonella* in Human Macrophages in a Host Cell Death-Independent Manner

**Figure 3:**
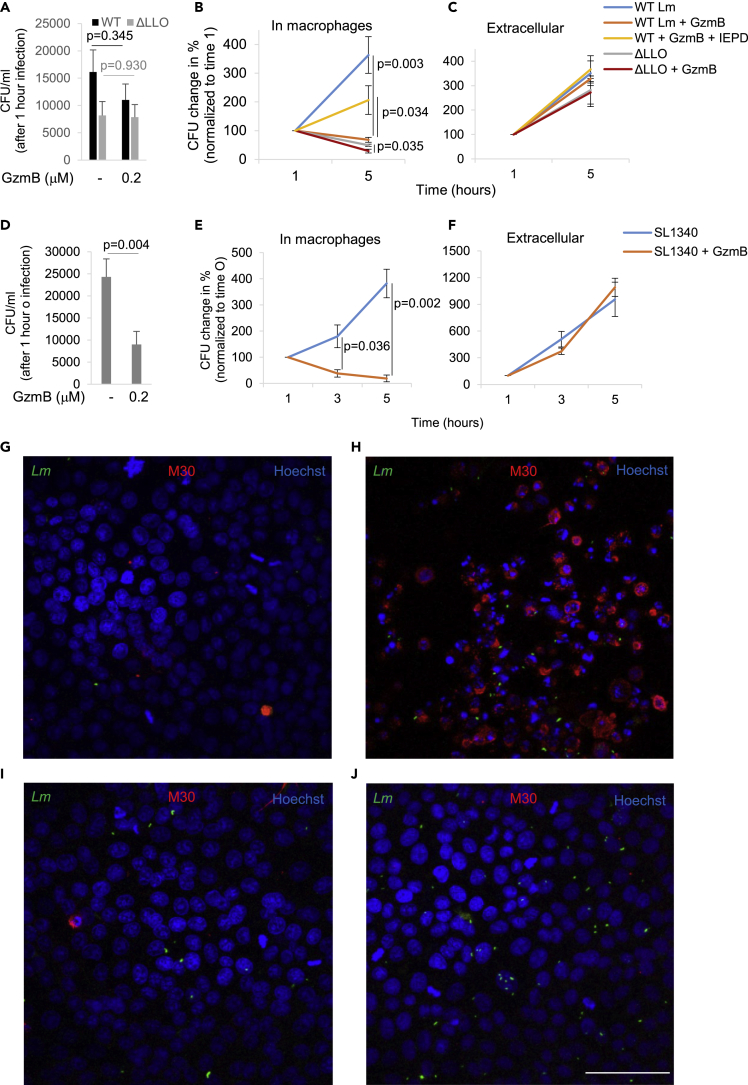
GzmB Treatment during Infection Inhibits Growth of Intracellular *Listeria* and *Salmonella* in Human Macrophages without the Induction of Host Cell Apoptosis Human macrophages were infected with wild-type (WT) or LLO-deficient Lm (ΔLLO) (A and B), as well as with *Salmonella* SL1344 (D and E), in the presence or absence of 0.2 μM GzmB. To indicated samples, GzmB was added together with its specific inhibitor Ac-IEPD-CHO. At indicated times after washing and removal of extracellular bacteria by gentamicin treatment, the macrophages were hypotonically lysed and the CFUs were enumerated on agar plates. The averages ± SEM of the CFU counts from four independent experiments after 1 h infection time is indicated in (A) and (D). To focus on intracellular growth, the CFU changes in four independent experiments were normalized to the 1-h time point (B) and (E). Extracellular bacteria were grown in infection medium ±0.2 μM GzmB for 1 h before being diluted in appropriate bacteria broth for continued growth for 4 h (C and F). HeLa cells were infected with CFSE-labeled *Listeria monocytogenes* in the presence of 0.2 μM GzmB (G), GzmB + sublytic perforin (PFN) (H), PFN only (I), or left untreated (J) and were then further cultured for 4 h before being fixed and stained with the CytoDeath M30 antibody and Hoechst (blue) for confocal microscopy. Scale bar is 50 μm. Representative images from independently repeated experiments are shown.

The microscopy experiments clearly suggested decreased bacterial load after infections in the presence of low-dose GzmB. However, for additional quantification, colony-forming unit (CFU) assays were necessary. For this purpose, human macrophages were infected with wild-type (WT) *Lm* or a strain lacking LLO (ΔLLO) at a multiplicity of infection of 10 in the presence or absence of GzmB or the unspecific protease, trypsin. At indicated times, the macrophages were hypotonically lysed and the bacterial CFUs enumerated on agar plates. CFU counts at the 1-h infection time point demonstrated no significant differences of the uptake of the *Lm* strains into the host cells (Figure 3A). This was in sharp contrast to *Lm* infections in the presence of trypsin that significantly reduced the initial *Listeria* load in macrophages (Figure S4A). However, most of the macrophages detached from the surface during the trypsin treatment, which might hamper bacterial uptake. After the initial infection, the presence of GzmB significantly reduced the intracellular growth of WT *Lm* toward the range of the CFU counts of the severely attenuated ΔLLO bacteria (Figure 3B). Again, this was different from trypsin treatment where the growth of the few intracellular bacteria was not completely inhibited but was significantly reduced (Figure S4B). GzmB even further reduced the growth of ΔLLO bacteria suggesting that protease affected additional virulence that contributes to intracellular bacterial survival (Figure 3B). This intracellular growth inhibition in macrophages in response to GzmB was clearly due to attenuated bacterial virulence, rather than some other unspecific growth defect, as extracellular bacteria were not affected by the protease (Figure 3C), and, remarkably, also not by trypsin (Figure S4C).

As several important SL1344 virulence factors were efficiently destroyed by GzmB, we also performed growth assessment in CFU assays for intracellular *Salmonella.* Interestingly, the presence of GzmB already significantly inhibited the initial uptake of *Salmonella* suggesting that factors involved in binding and initial uptake might be particularly well targeted by the immune protease (Figure 3D). In addition, the intracellular growth of *Salmonella* in human macrophages was severely crippled in response to GzmB (Figure 3E). Again, this growth inhibition was observed only within host cells and extracellular *Salmonella* grew normally upon GzmB treatment (Figure 3F).

Confusingly, listeriolysin O was demonstrated to be capable of delivering the granzymes into target cells to induce cell death (Browne et al., 1999). Therefore, we assessed caspase activation in response to *Lm* infection and GzmB using the highly sensitive M30 CytoDEATH antibody that detects caspase-cleaved cytokeratin 18 early in apoptosis (Leers et al., 1999). Infections in the presence of GzmB induced no caspase activation (Figure 3G) as compared with infection only (Figure 3J). However, when we infected in the presence of GzmB and the efficient delivery protein perforin (PFN), we observed high caspase activity in almost all the cells after 4 h (Figure 3H). The PFN dose used was clearly sublytic as PFN-only treatment did not affect the host cells (Figure 3J). This striking difference might be explained by the use of recombinant LLO in the earlier study at concentrations that might not be reached by the endogenous virulence factor, in particular if GzmB degrades this factor upon secretion. Also, the simultaneous infection of these target cells might alter the susceptibility to lytic proteins by an increased membrane repair response as already observed in human dendritic cells upon *Lm* infections (Walch et al., 2007).

However, our data confirm the published results that, for efficient bacterial killing, without the support of bystander cells, GzmB needs cytosolic delivery by GNLY (Dotiwala et al., 2017, Walch et al., 2014). Moreover, these results additionally indicate that GzmB in a GNLY-independent manner attenuates bacterial virulence to help bystander cells eliminating the engulfed bacteria.

### The Human Natural Killer Cell Line YT Indy Restricts the Growth of Intracellular Bacteria in a Gzm-Dependent Manner

**Figure 4:**
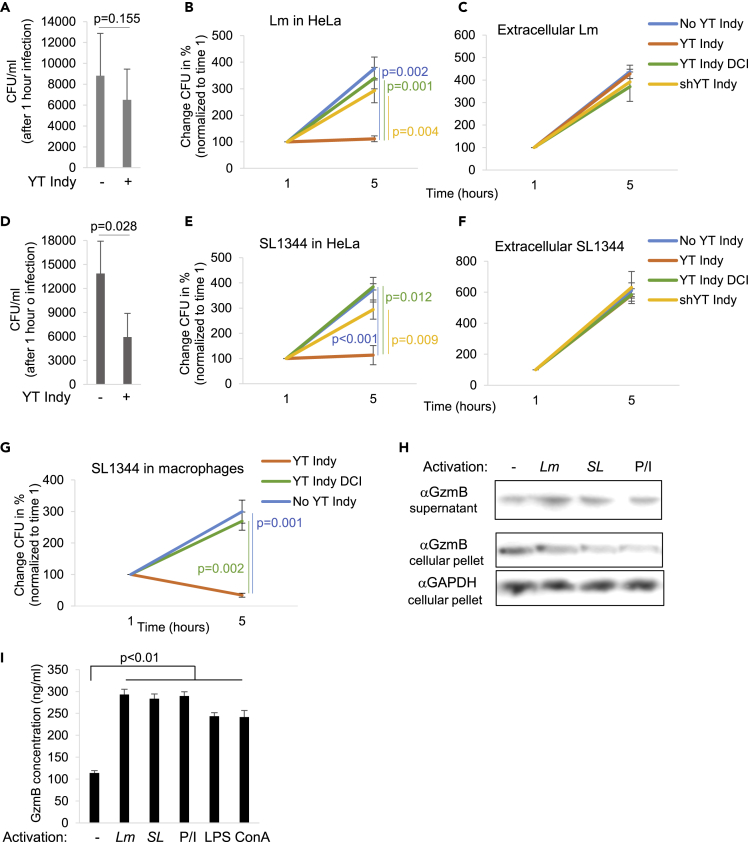
The Human NK Cell Line, YT Indy, Restricts the Growth of Intracellular Bacteria in a Gzm-Dependent Manner HeLa cells or human primary macrophages were infected with *Lm* (A and B) or with *Salmonella* SL1344 (D, E, and G) in the presence or absence of the natural killer cell line YT Indy. In indicated experiments, the YT Indy cells were pretreated with the serine protease inhibitor DCI. In other control experiments, an YT Indy clone (shYT Indy) was used that was silenced for GzmB and GzmH by shRNA transduction (see Figures S5A and S5B). After washing and removal of extracellular bacteria by gentamicin treatment, the infected cells were at indicated times hypotonically lysed and the CFUs were enumerated on agar plates. CFU counts of four independent experiments after 1 h of infection are plotted in (A) and (D). The CFU changes, normalized to the 1-h time point, are indicated in (B) and (E). Extracellular bacteria were grown in the infection medium in the presence or absence of YT Indy cells for 1 h and then diluted in appropriate bacterial broth for a continued incubation of 4 h (C and F). In (H), GzmB secretion from YT Indy cells after 4 h of co-incubation was assessed in immunoblots of the cellular supernatants using an anti-GzmB antibody. GzmB and GAPDH in the cellular pellet served as loading controls. Representative blots from independently repeated experiments are shown (see also Figure S5C) The supernatants of two independent experiments were additionally analyzed by GzmB ELISA (I). As positive control activations during these assays served PMA/Ionomycin (P/I), lipopolysaccharide (LPS), and concanavalin A (ConA) treatment. For all CFU assays, averages ±SEM are presented.

*Lm* and *Salmonella* upon infection first enter intestinal epithelial cells (Lorkowski et al., 2014, Pizarro-Cerda et al., 2012). Within the epithelial layer (Mayassi and Jabri, 2018) and in the lamina propria underneath (Fuchs and Colonna, 2011), there are plenty of lymphocyte subsets, including γδ T cells, that potentially secrete GzmB (Ogata and Itoh, 2016). To test an experimental model that roughly mimics this initial invasion phase, we infected the human epithelial cell line, HeLa, in the presence of the natural killer (NK) lymphocytic line, YT Indy. YT Indy is a highly cytolytic human cell line that expresses vast amounts of GzmB and GzmH but not GzmA, GzmK, or GzmM (Su et al., 1994). To do so, we infected HeLa cells with WT *Lm* (Figures 4A and 4B) or SL1344 (Figures 4D and 4E) at an MOI of 10 in the presence or absence of the NK cells at an effector cell:bacteria ratio of 10. We then removed NK cells and non-internalized bacteria by washing and gentamicin treatment, which kills the remaining extracellular bacteria. After the infection, we monitored the initial bacterial load (Figures 4A and 4D) and intracellular bacterial growth (Figures 4B and 4E) by CFU assays. In contrast to the no effector cell control, Lm and SL1344 failed to grow in HeLa cells when infected in the presence of YT Indy cells. We observed a bacteriostatic effect in HeLa cells mediated by the YT Indy cells, potentially due to the limited bactericidal mechanisms of epithelial cells. This was in contrast to the impact of YT Indy cells on the growth of SL1344 in human macrophages, where we observed a decrease of the intracellular bacteria load (Figure 4G). YT Indy cells had no apparent effect on the viability of extracellular bacteria (Figures 4C and 4F). More importantly, pre-treatment of the killer cells with the serine protease inhibitor 3,4-dichloroisocoumarin (DCI) or the use of a YT Indy line (shYT Indy), silenced for GzmB and GzmH (Figures S5A and S5B) that we have recently established (Chiusolo et al., 2017), abolished their impact on intracellular bacterial growth, suggesting involvement of the serine protease GzmB in this growth inhibition (Figures 4B and 4E). Additionally, in support of the Gzms to be responsible for the growth inhibition was the observation that YT Indy cells secrete GzmB upon contact with bacteria as demonstrated by immunoblot (Figure 4H) and ELISA (Figure 4I). The GzmB secretion was at the same level as induced by the commonly used positive control treatments, such as PMA/Ionomycin (P/I), concanavalin A (ConA), and lipopolysaccharide (LPS) suggesting activation of pattern recognition receptors by the bacterial contact and subsequent calcium signaling (Hoving et al., 2014) as the trigger for the observed GzmB secretion. Importantly, the GzmB release was unaffected by the pretreatment with DCI (Figure S5C), indicating that the killer cells were not impaired by the serine protease inhibitor pretreatment and secrete most likely proteolytically inactivated enzymes. Together these data again suggest that the NK cell-derived Gzms cripple bacterial virulence, subsequently causing intracellular growth inhibition.

### Short-Term Activated Primary Killer Lymphocytes Restrict the Growth of Intracellular Bacteria

**Figure 5:**
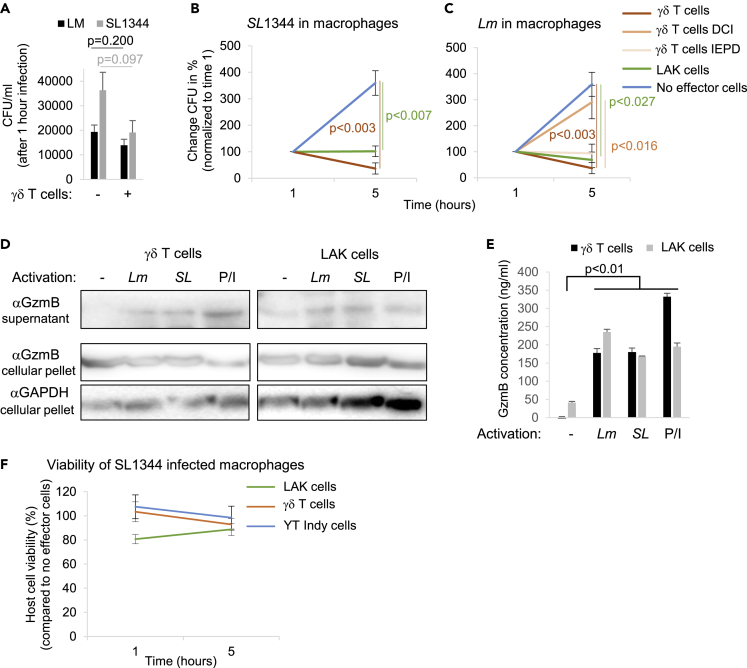
Short-Term Activated Primary Killer Lymphocyte Restrict the Growth of Intracellular Bacteria by the Attenuation of Virulence (A–F) (A), Human macrophages were infected with *Salmonella* SL1344 or *Lm* in the presence or absence of indicated killer cells. For indicated experiments, the γδ T cells were pretreated with DCI or the co-incubation was performed in the presence of the inhibitor Ac-IEPD-CHO. After removal of extracellular bacteria by PBS wash and gentamicin treatment, the infected cells were hypotonically lysed at indicated times and the CFU assessed on agar plates. CFU counts of three independent experiments after 1 h of infection are shown in (A). The CFU changes, normalized to the 1-h time point, are demonstrated in (B) and (C). GzmB secretion from indicated killer cells into the supernatants was assessed by GzmB immunoblots (D), as well as by GzmB ELISA (E). As positive control activation during these assays served PMA/Ionomycin (P/I) treatment. GzmB and GAPDH in the cellular pellet served as loading controls. Representative blots from independently repeated experiments are shown (see also Figure S6B) In (F), BCECF-AM-prelabeled macrophages were infected with SL1344 ± indicated killer cells. After washing and removal of extracellular bacteria by gentamicin treatment, at indicated times the released fluorescence in the supernatant of three independent experiments was assessed by plate reader and normalized to the no effector cell control to calculate host cell viability. For all data plots, averages ±SEM are presented.

To further challenge our hypothesis, we asked if short-term activated primary lymphocytes may restrict bacterial virulence and, with that, intracellular growth. As model cells, we chose lymphokine-activated killer (LAK) as well as γδ T cells. Both cell models were demonstrated to display potent cytolytic activity *in vitro* (Rayner et al., 1985), the latter also in the context of bacterial infections (Zheng et al., 2013). Therefore, we infected human macrophages with SL1344 (Figures 5A and 5B) and *Lm* (Figures 5A and 5C) at an MOI of 10 in the presence of indicated primary killer cells at an effector:bacteria ratio of 10. We found an impaired bacterial uptake in the presence of γδ T cells that fell just short of statistical significance (Figure 5A). However, intracellular bacterial growth was significantly reduced in response to the killer cells (Figures 5B and 5C). This bacterial growth restriction was not explained by direct inhibition of extracellular bacteria that were not affected by the killer cells (Figure S6A). Pretreatment of γδ T cells with DCI, but not the presence of the GzmB inhibitor Ac-IEPD-CHO, significantly reduced their impact on intracellular *Lm* growth (Figure 4C), suggesting that various granzymes might contribute to the virulence attenuation. As for YT Indy cells (Figures 4H and 4I), we found an induction of the GzmB secretion in the supernatants from both cell types in response to the bacteria (Figures 5D, 5E, and S6B). As observed in infections assays in the presence of purified GzmB (Figures 3G–3J), the impaired intracellular bacterial growth was not caused by enhanced death of the infected host cells that were unaffected by the presence of killer cells during the infection procedure (Figure 5F).

### γδ T Cells and GzmB Inhibit the Growth of *Mycobacterium bovis* BCG in Human Macrophages in a TNF-Alpha-Independent Manner

**Figure 6:**
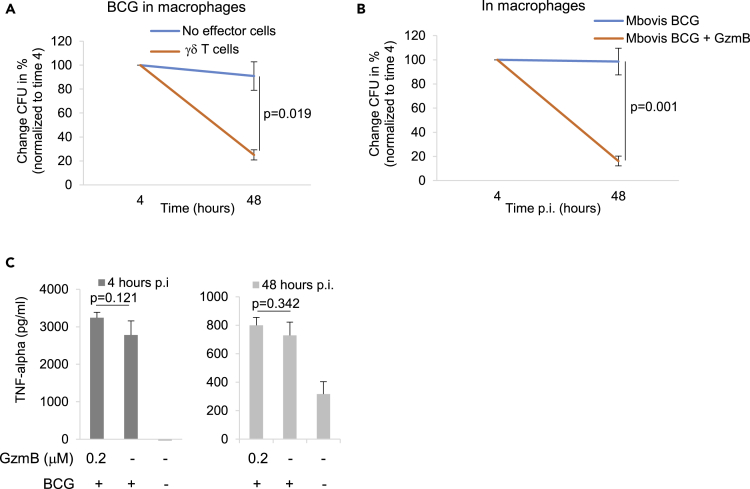
γδ T Cells and GzmB Inhibit the Growth of *Mycobacterium bovis* BCG in Human Macrophages in a TNF-Alpha Independent Manner (A and B) Human macrophages were infected for 4 h with *Mycobacterium bovis* BCG ±γδ T cells (A) or ±0.2 μM GzmB (B). After removal of extracellular bacteria, the infected cells were hypotonically lysed at indicated times and the CFUs of three independent experiments were counted. Averages ±SEM are presented. (C) TNF-α levels in the supernatant of BGC-infected macrophages ±0.2 μM GzmB directly after the infection (left panel) or after wash and removal of extracellular bacteria 2 days post infection (right panel) were measured by ELISA. Data of independently repeated experiments are presented as average ± SEM.

A recent study observed mycobacterial growth inhibition in human macrophages that was mediated by GzmA secretion from γδ T cells (Spencer et al., 2013). We also infected human macrophages with *Mycobacteria bovis* BCG in presence of γδ T cells and could indeed recapitulate the intracellular bacterial growth inhibition by CFU assays (Figure 6A). However, the cited study speculated about the underlying mechanism of the observed growth inhibition to be mediated by differential tumor necrosis factor alpha (TNF-α) expression that was driven by GzmA. Therefore, we also infected macrophages with *Mycobacteria bovis* BCG in the presence or absence of GzmB and measured intracellular bacterial growth by CFU assays and TNF-α secretion by ELISA. Although GzmB treatment during BCG infection of human macrophages impaired intracellular bacterial growth (Figure 6B), we could not detect significant differences in TNF-α levels in GzmB exposed versus non-exposed culture supernatants (Figure 6C). In our experimental model, TNF-α secretion was essentially driven by the bacterial infection and was not further enhanced by GzmB treatment.

### Expression of a GzmB Uncleavable LLO Mutant Rescued *Listeria* Virulence upon GzmB Treatment

**Figure 7:**
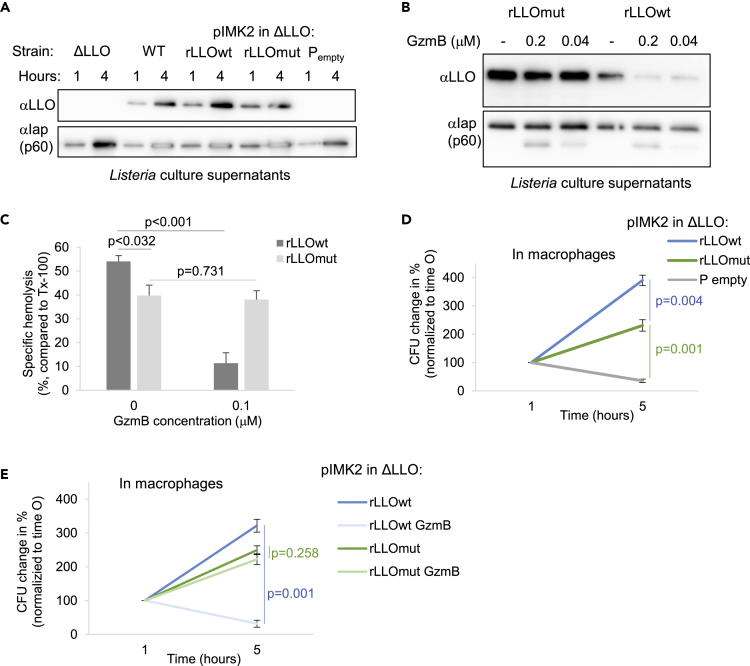
Expression of a GzmB Uncleavable LLO Mutant Rescued *Listeria* Virulence upon GzmB Treatment (A and B) Culture supernatants of wild-type (WT) or listeriolysin O-deficient (ΔLLO) *Listeria*, transfected with indicated pIMK2 constructs, were primed for 1–4 h (A) or treated with GzmB for 20 min (B). Secretion performance over time and GzmB cleavage of the proteins were assessed by anti LLO and anti Iap immunoblots. (C–E) (C) *Listeria*-primed supernatants were pretreated with indicated concentrations of GzmB for 20 min as in Figure 2A before treating human red blood cells for 15 min at 37° C. Hemoglobin release was measured spectrophotometrically at 405 nm wavelength. Indicated *Listeria* mutants were infected in the absence (D) or presence of 0.2 mM GzmB (E) before the intracellular bacterial load was assessed at indicated time points by CFU assay. Averages ±SEM are presented.

To establish a causative link between a GzmB-mediated cleavage event and the observed decrease in virulence, we generated GzmB-uncleavable factor mutants (Martinvalet et al., 2008, Walch et al., 2014). The most promising candidate for the generation of an uncleavable mutant was LLO, as this mediator is essential for *Lm* virulence (Hamon et al., 2012), it has membranolytic activity that is easily assessed, and it is cleaved by GzmB with remarkably high efficiency. Analysis of GzmB cleavage sites using an N-terminal GST-LLO fusion protein by SDS-PAGE and Coomassie staining, as well as immunoblotting, revealed three dominant cleavage fragments (Figure S7). The GzmB cleavage sites were identified to be D193, D200, and D416 by N-terminal sequencing, as well as by mass spectrometry analysis. After site-directed mutagenesis of these three aspartic acids into alanines, we integrated either wild-type LLO (rLLOwt) or the uncleavable mutant LLO (rLLOmut) into the chromosome of the LLO-deficient Lm strain (ΔLLO), using the *Listeria* integration vector pIMK2 (Monk et al., 2008). Analysis of listerial culture supernatants indicated a similar secretion performance of the recombinant LLO proteins as that of wild-type LLO (Figure 7A). GzmB treatment of listerial supernatant demonstrated that, at a given GzmB concentration (0.2 μM) and exposure time (20 min), the uncleavable variant of LLO was protected from degradation, whereas Iap was still cleaved (Figure 7B). Although the hemolytic activity of mutated recombinant LLO was somewhat, yet significantly, reduced compared with the wild-type form of the protein, it was not further impaired by the pre-treatment with GzmB (Figure 7C). The strain expressing mutated recombinant LLO also displayed a significant growth defect in human macrophages as compared with the wild-type version (Figure 7D). This reduced enzymatic activity of the mutated protein was likely due to the replacement of three aspartic acids with alanines that changes the charge of the hemolysin at the given pH. However, Lm expressing the uncleavable variant of the hemolysin grew significantly better as the line that integrated the empty vector only (p_empty_ in Figure 7D), rendering the mutated line a valuable tool to test virulent growth in response to GzmB. Indeed, when we infected human macrophages with these bacterial strains in the presence of low-dose GzmB, the growth of the listerial line expressing uncleavable mutant LLO was not further affected by GzmB (Figure 7E), indicating that the GzmB-mediated degradation of this virulence factor caused the observed intracellular *Listeria* growth inhibition.

## Discussion

In this study, we present compelling evidence that virulence-related external proteins from *Listeria monocytogenes*, *Salmonella typhimurium*, and *Mycobacteria tuberculosis* are more efficiently targeted and destroyed by GzmB, compared with randomly picked cytosolic control proteins. The degradation of virulence factors can also be mediated by unspecific proteases, such as trypsin; the collateral tissue damage would be potentially much more dramatic compared with highly specific proteases, illustrated by non-discriminating digestion of all tested proteins by trypsin, including GST.

Virulence is a toolbox of mostly secreted or externally exposed factors that evolved in pathogenic bacteria to establish infections (Sparling, 1983, Webb and Kahler, 2008). Virulent *Listeria, Salmonella*, and *Mycobacteria* use secreted or membrane-bound adhesion factors, called internalins (Inl), *Salmonella* invasion proteins (Sip), or mycobacterial adhesins, such as HbhA, and many others to bind and invade phagocytes and non-phagocytic cells, such as epithelial cells (Bierne et al., 2007, Ibarra and Steele-Mortimer, 2009, Squeglia et al., 2018). After uptake, *Listeria* escape the endosomes/phagosomes using membranolytic toxins, LLO and PlcA + PlcB, to avoid degradation in the lysosomal compartment (Cossart, 2011). *Salmonella* employ factors that impact on positioning of the phagosome, such as SipC and SopE, to avoid phagosome maturation and lysosomal fusion (Forrellad et al., 2013, Jantsch et al., 2011). Additionally, *Salmonella* and *Mycobacteria* can also release radical degrading enzymes, such as AhpC, the caseinolytic proteases (Clp), or catalase, to resist low pH and oxidative and nitrosative stress in the harsh environment of the phagosomes (Forrellad et al., 2013, Ibarra and Steele-Mortimer, 2009). Ultimately, these mechanisms enable the facultative intracellular bacteria to escape the hostile environment in the extracellular space where a multitude of innate and adaptive immune defense mechanisms can potentially harm bacterial pathogens. Simultaneously, virulence factors of these facultative intracellular bacteria promote prolonged survival in specialized immune cells by inhibiting their bactericidal mechanisms, the cell death machinery (Robinson and Aw, 2016), or the instruction of an adaptive immune response (Zhai et al., 2019). Considering the crucially important functions of these factors, it seems highly likely that the degradation of several of these proteins would severely cripple intracellular growth of inflicted bacteria.

Therefore, from a host perspective, it would seem like an efficient counterstrategy to target specifically these virulence mediators that are visible and potentially vulnerable to host defense mechanisms. Indeed, the immune system evolved various mechanisms to fight bacterial virulence. Secreted immunoglobulin A (IgA) interferes with bacterial invasion and adhesion by targeting surface exposed factors (Lamm, 1997), in addition to triggering the complement cascade to eliminate the pathogens (Heesterbeek et al., 2018). Pattern recognition receptors (PRRs) bind bacterial endotoxins and other surface exposed factors to mount an inflammatory response and to alarm the host defense (Heumann and Roger, 2002). Many acute phase proteins directly counteract bacterial siderophores by the sequestration of iron during infections (Parrow et al., 2013).

Immune proteases are crucial for antibacterial defense. Neutrophil elastase and cathepsin G are indispensable for the elimination of engulfed pathogens, probably in cooperation with microbicidal peptides and oxygen radicals produced by the NADPH oxidase (Korkmaz et al., 2008). How these innate immune proteases actually kill bacteria was not well defined. However, proteases may directly interfere with bacterial virulence as they can degrade bacterial factors and consequently attenuate bacterial virulence and pathogenicity.

We recently discovered that the granzymes rapidly kill bacteria when delivered into the bacterial cytosol by the antimicrobial protein GNLY (Walch et al., 2014). This rapid killing was triggered by the proteolytic degradation of vital bacterial proteins, such as the components of the respiratory chain, radical oxygen degrading enzymes, and the protein synthesis machinery (Dotiwala et al., 2017, Walch et al., 2014). However, to induce this death pathway, the two cytotoxic effector molecules have to act in a highly coordinated manner. As GNLY is expressed only in activated lymphocytes (Krensky and Clayberger, 2009), this exclusive cooperation is predominantly given during a cytotoxic lymphocyte attack against bacterial infected target. To do so, cytotoxic lymphocytes need to recognize and bind infected target cells to release the two effectors together with yet another pore-forming protein, perforin, to primarily shuttle GNLY and GzmB into the infected target cell (Froelich et al., 1996). Specific cytotoxic lymphocyte attacks are core features of adaptive immunity. They mount on established bacterial infections and are time consuming.

In the present study, we reveal a synergistic antibacterial activity of the granzymes during innate immunity that acts at the starting point of an infection by targeting bacterial virulence. We present here a function of extracellular Gzms that is independent of GNLY-mediated delivery into the bacterial cytosol. GzmB cleaves with remarkably high efficiency secreted bacterial virulence mediators, such as *Listeria* LLO and *Salmonella* SipA, SipC, and FljB. The efficient proteolytic degradation of these crucially important bacterial factors by GzmB is corroborated by numerous functional assays, demonstrating impaired *Listeria* phagosomal escape, drastically decreased *Salmonella* load early in the infection, as well as impaired intracellular bacterial growth in epithelial cells and macrophages.

Interestingly, in agreement with our findings, these virulence molecules during the infection of neutrophils are also efficiently degraded by innate immune proteases, such as the neutrophil metalloproteinase-8 (Arnett et al., 2014) and neutrophil elastase (Weinrauch et al., 2002), respectively, highlighting the evolutionary relevance of this host-pathogen interactions. More recently, it was demonstrated that neutrophils in response to *Mycobacteria* infection up-regulate *in vivo* GzmB (Mattila et al., 2015) that might also contribute to bacterial killing by targeting virulence as GNLY is not co-expressed. Our results reveal an unexpected function of innate cytotoxic lymphocytes exerted by the release of the granzymes in limiting the bacterial cellular entry as well as in impairing bacterial intracellular growth in established infections. The inhibition of intracellular bacterial growth was not explained by increased cell death or by the increased secretion of proinflammatory cytokines, such as TNF-a, from the host cells in the presence of extracellular granzymes or killer lymphocytes.

Exemplified with the major *Listeria* virulence mediator LLO, we conclusively demonstrate that GzmB destroys the membranolytic function of this factor, which is a prerequisite of the phagosomal escape. Indeed, when infected in the presence of GzmB, *Listeria* fail to multiply in macrophages and do not recruit the actin cytoskeleton. Importantly, the generation of an uncleavable form of LLO rescues hemolytic activity and intracellular replication in the presence of GzmB, causatively linking a single Gzm-mediated virulence factor destruction with impaired virulent behavior in host cells.

Mechanistically, we demonstrate in co-uptake assays with fluorescently labeled GzmB that the positively charged protease binds to the negatively charged bacterial surface to be co-endocytosed into host cells. The co-endocytosis will increase the GzmB concentration in the bacteria-containing intracellular compartment where the protease continues to degrade externalized virulence factors and therefore to impair virulence. The probably less efficient co-endocytosis of uncharged trypsin might therefore explain our result that the presence of trypsin during the infection impairs intracellular *Listeria* growth less efficiently than GzmB, although it is proteolytically more active in cleavage assays.

Overall, in a more physiological context, we think that this immune mechanism might be important at mucosal surfaces where intraepithelial lymphocytes and potentially other cell types secrete GzmB in response to bacterial invaders or inflammatory stimuli (Olivares-Villagomez and Van Kaer, 2018). Extracellular GzmB will then degrade external virulence mediators to disarm the invading bacterial pathogens and subsequently enable bystander immune and non-immune cells to eliminate the bacteria.

### Limitations of the Study

In this work, we reveal an antimicrobial function of GzmB by efficiently destroying secreted bacterial virulence mediators under *in vitro* conditions. We hypothesize that this antimicrobial mechanism might be particularly important at mucosal surfaces where invading bacteria might trigger the release of Gzms by intraepithelial lymphocytes (IELs) or lamina propria cells and thus limit bacterial virulence and further spread. A major limitation of this study is therefore the absence of any *in vivo* proof. There are five Gzms in humans and even 10 Gzms in mice with overlapping substrate specificities; therefore, single gene knockdown will likely have no effect. However, a mouse model deficient for the major Gzms expressed in IELs could be challenged orally with bacteria to assess their invasiveness and ability to induce systemic disease. Depletion of IELs in mice was already demonstrated to impair *Salmonella* clearance (Li et al., 2012); however, the underlying mechanism was not solved in this study. Another question that remains unanswered is what happens to extracellular bacteria, such as *Staphylococcus aureus*, *Pseudomonas aeruginosa*, or pathogenic *E. coli* (EPEC). Could the Gzms also attenuate the virulence of extracellular bacteria and therefore increase their susceptibility to defense mechanisms, such as complement lysis or the lethal engulfment by phagocytes?

## Methods

All methods can be found in the accompanying Transparent Methods supplemental file.

## Supplemental Information

Document S1. Transparent Methods and Figures S1–S7
