# Supplementary material for: Granzyme B Attenuates Bacterial Virulence by Targeting Secreted Factors
Source: iScience. 2020 Feb 21;23(3):100932. doi: 10.1016/j.isci.2020.100932 (PMC7063247; doi:10.1016/j.isci.2020.100932)
Supplement: Document S1. Transparent Methods and Figures S1–S7 [file mmc1.pdf]

iScience, Volume *23*

Supplemental Information

Granzyme B Attenuates Bacterial

Virulence by Targeting Secreted Factors

Diego López León, Patricia Matthey, Isabelle Fellay, Marianne Blanchard, Denis Martinvalet, Pierre-Yves Mantel, Luis Filgueira, and Michael Walch

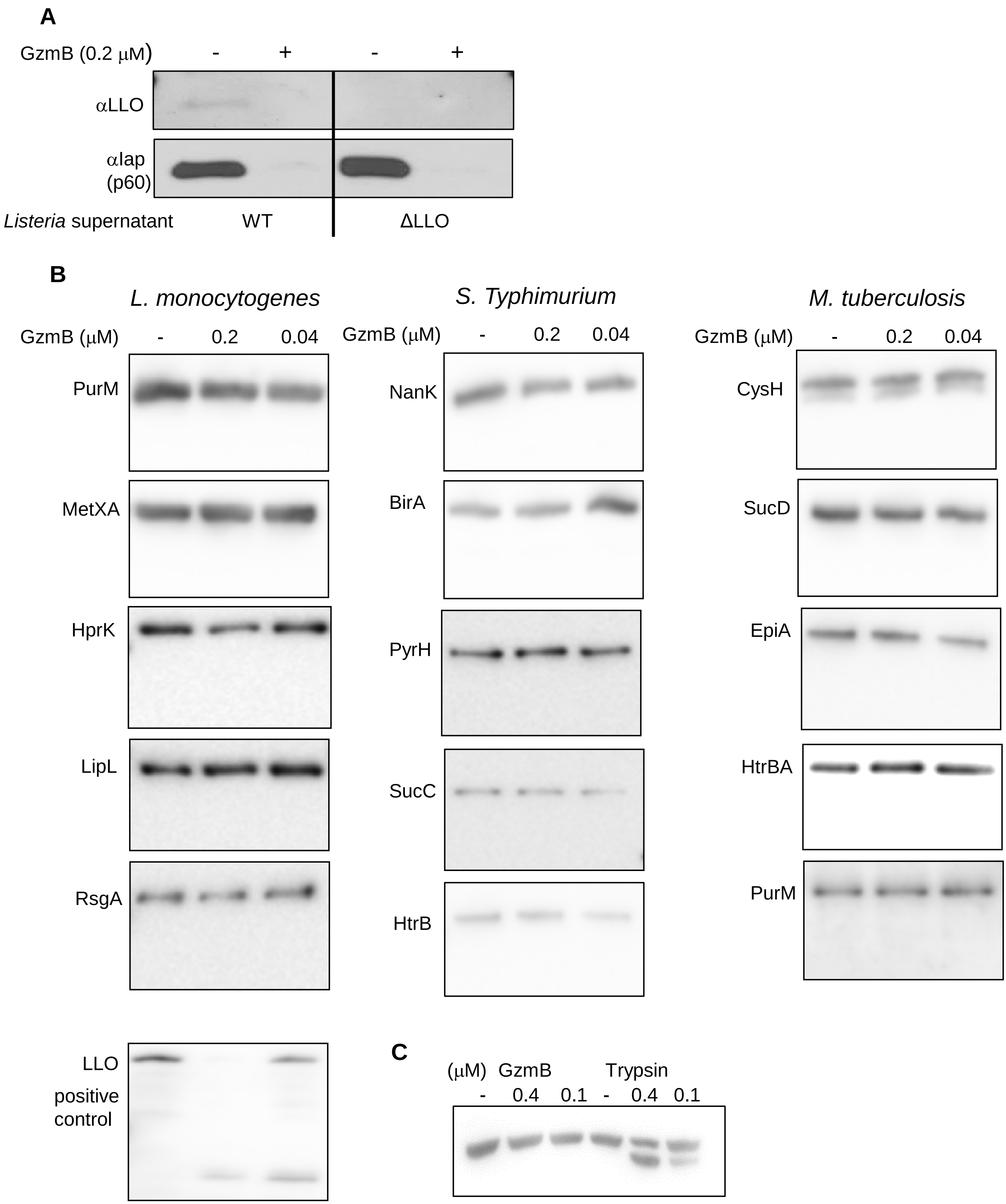

Fig S1. GzmB targets bacterial effectors but not cytosolic proteins. Related to Figure 1. (**A**) Culture supernatants of wild-type (WT) or listeriolysin O deficient (ΔLLO) *Listeria* were treated with GzmB for 60 minutes. Cleavage was assessed by αLLO and αlap immunoblots. (**B**), *E. coli* expressing GST-tagged *Listeria monocytogenes*, *Salmonella Typhimurium* or *Mycobacteria tuberculosis* randomly picked cytosolic control proteins were treated with lysozyme and then hypotonically lysed by freeze-thawing. Lysates were treated with indicated doses of GzmB for 20 minutes before substrate cleavage was assessed by GST immunoblot. As an additional control,

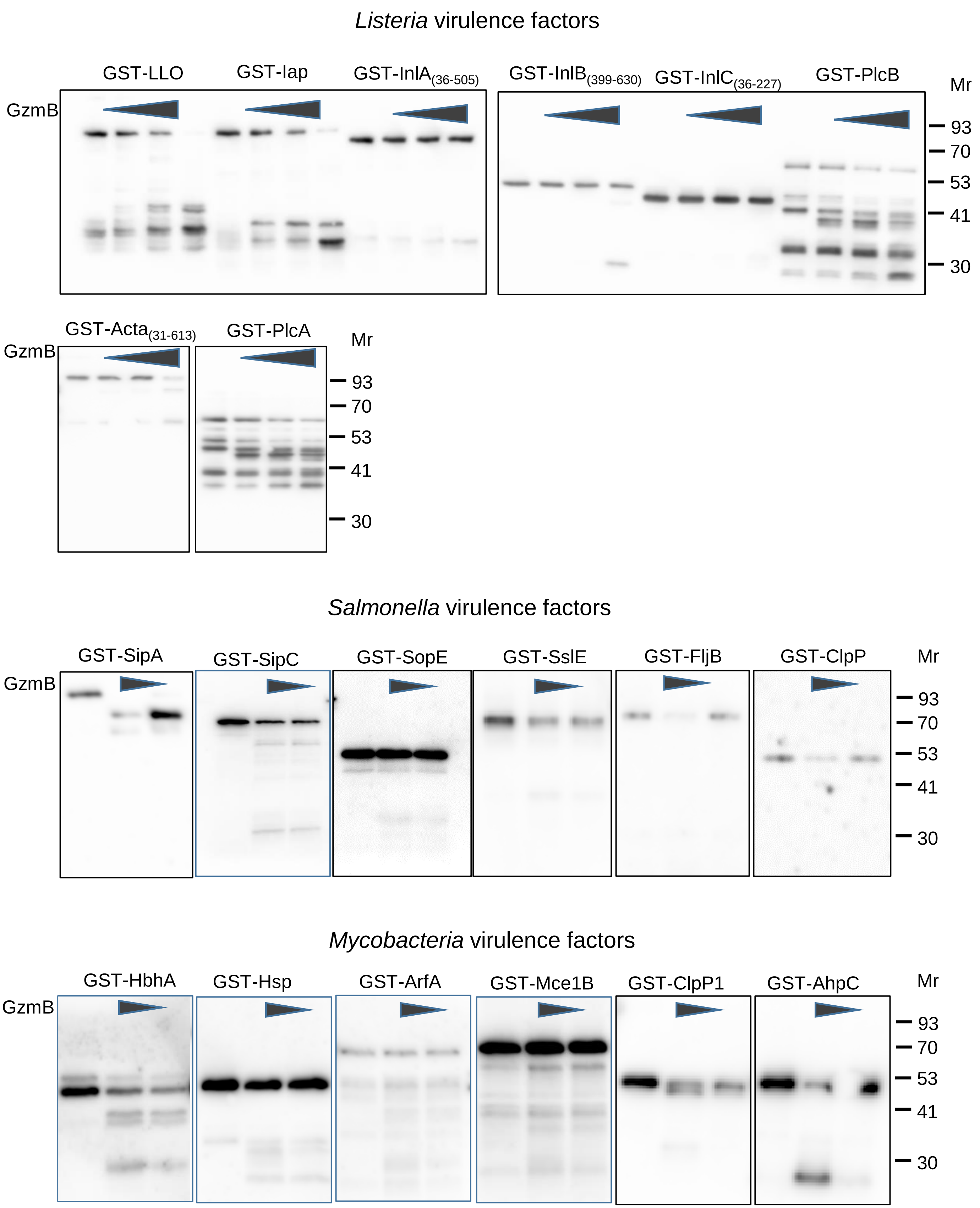

Fig S2. GzmB targets bacterial effectors, full lanes. Related to Figure 1.

The full lanes of the cleavage assay GST immunoblots of Figure at medium exposure times are demonstrated. For the virulence factors that were only partly cloned and expressed, the numbers of the flanking amino acids are given in parenthesis.

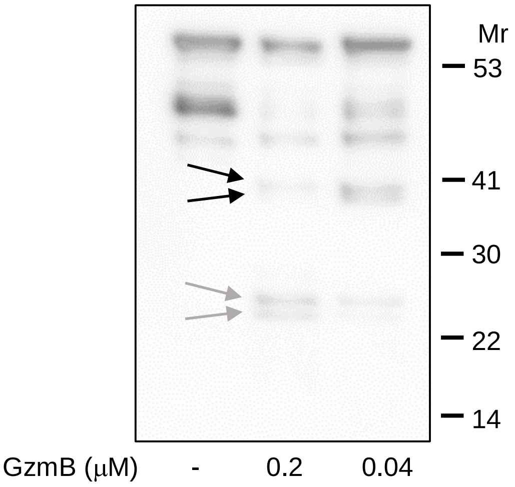

Fig S3. GzmB cleaves native LLO in *Listeria* supernatant. Related to Figure 2.

The high sensitive ECL reagent and a very long exposure time (> 10 minutes) of an αLLO immunoblot reveals the dominant C-terminal (black arrows) and N-terminal cleavage fragments (grey arrows) in *Listeria* supernatant after GzmB treatment.

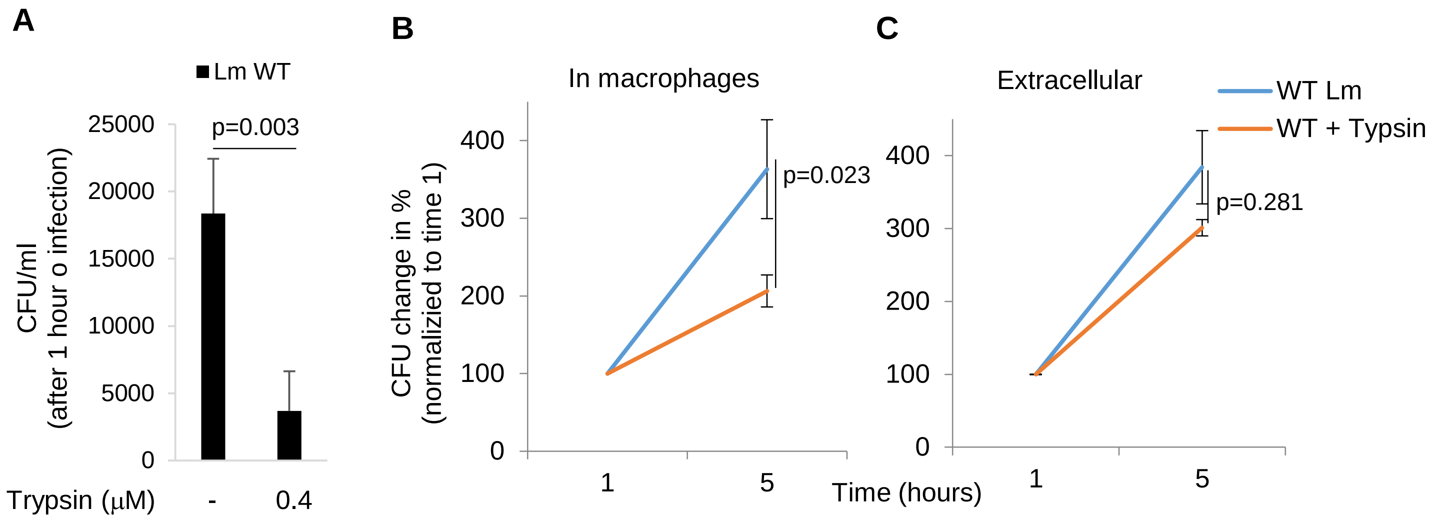

Fig S4. Trypsin treatment during infection inhibits uptake and growth of intracellular *Listeria* in human macrophages. Related to Figure 3.

Human macrophages were infected with wild-type (WT) Lm in presence or absence of 0.2 μM trypsin. After washing and removal of extracellular bacteria by gentamicin treatment, the macrophages were hypotonically lysed at indicated times and the CFU were counted on agar plates. The average+/-SEM of the CFU counts after 1 hour infection time is indicated in **A**. CFU changes are normalized to 1-hour time point in **B and C**. Extracellular bacteria were grown in infection medium +/- 0.2 μM Trypsin for 1 hour before being diluted in bacteria broth for continued growth for 4 hours (**C**).

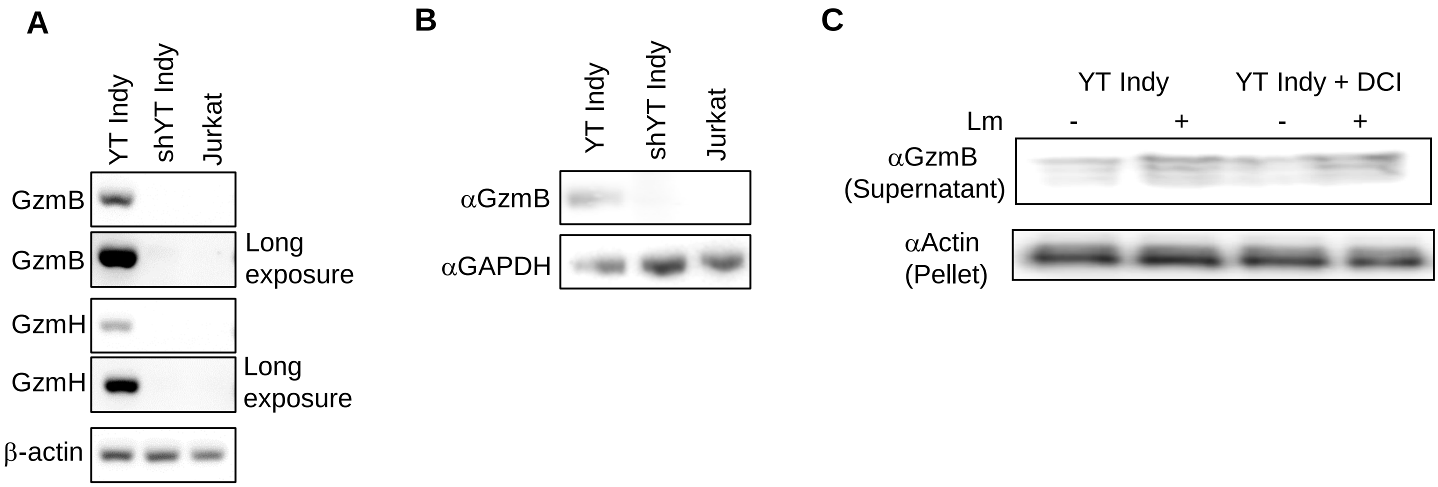

Fig S5. shYT Indy contain only minimal amounts of GzmB and no GzmH. YT Indy cells secrete GzmB after DCI pretreatment. Related to Figure 4.

GzmB and GzmH transcription as well as GzmB protein expression in shYT Indy was assessed in RT-PCR (**A**) and GzmB immunoblot (**B**), respectively, and compared to the parental WT YT Indy line as well as to Jurkat cells. β-actin transcript and GAPDH protein served as loading controls. (**C**), GzmB secretion from YT Indy cells, pretreated with DCI or not, after 4 hours of co-incubation was assessed in the supernatant by GzmB immunoblot. Actin in the cellular pellet served as loading control.

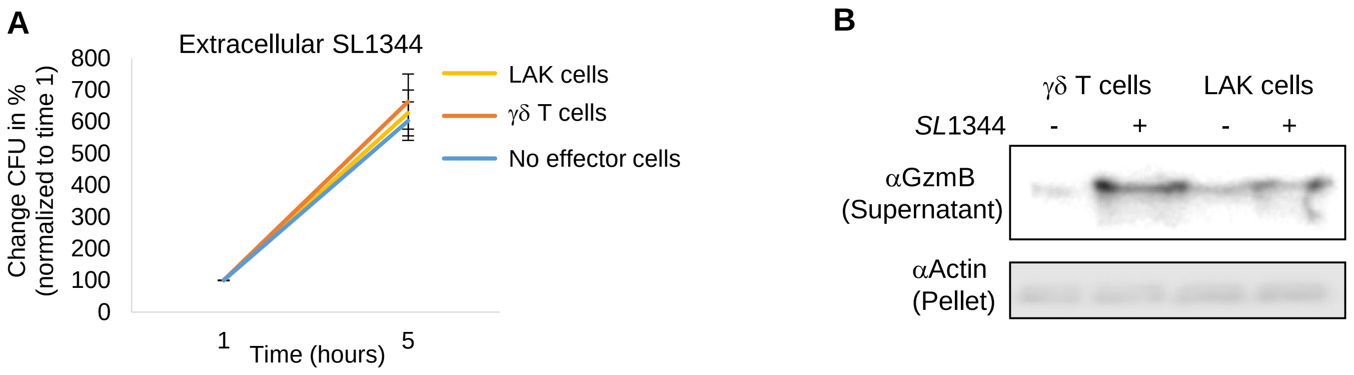

Fig S6. Primary killer cells do not affect extracellular bacteria and secrete GzmB upon co-incubation with bacteria. Related to Figure 5.

(**A**), Extracellular SL1344 were grown in infection medium +/- killer cells before dilution broth for continued growth for 4 hours and enumeration by CFU assays. (**B**), GzmB secretion from indicated killer cells after 4 hours of co-incubation was assessed by GzmB immunoblot of the cellular supernatant. Actin in the pellet served as loading control.

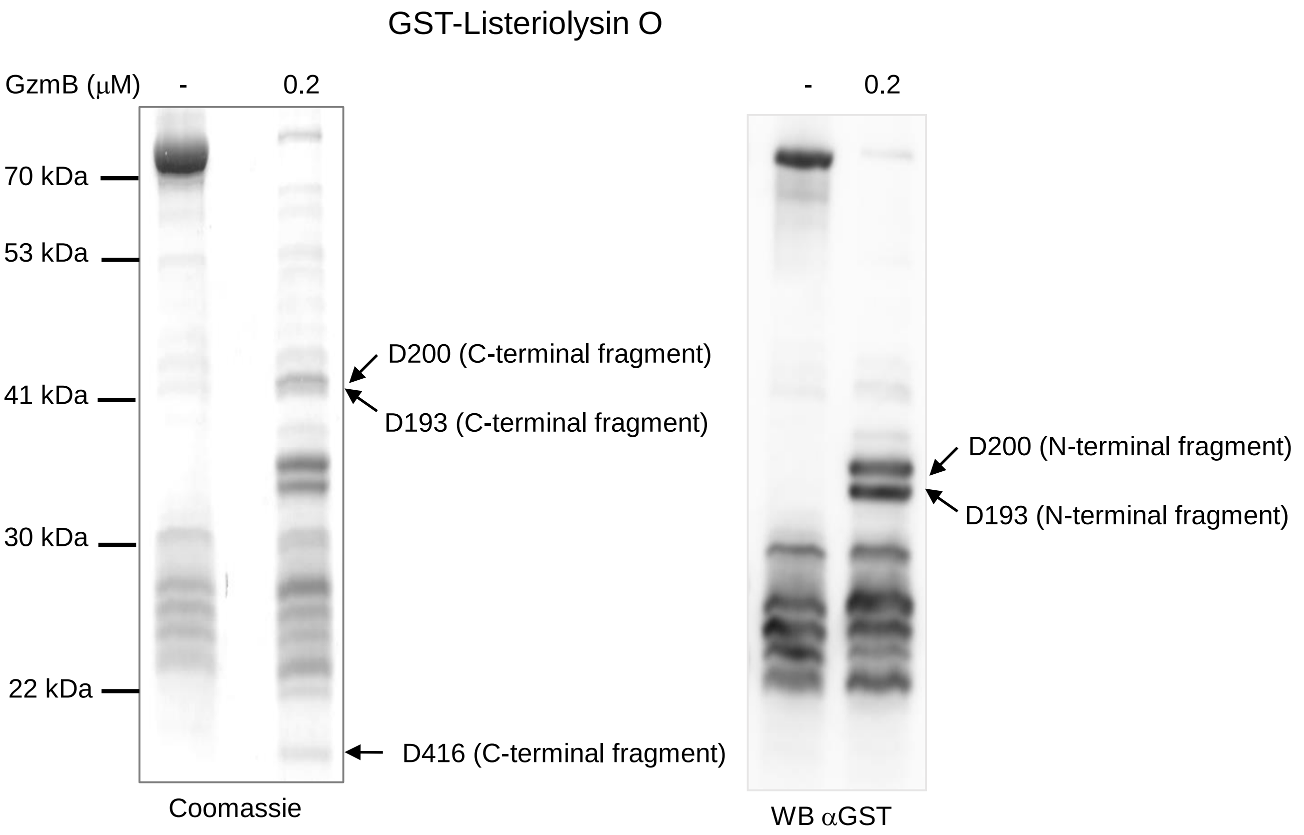

Fig S7. GzmB cleaves LLO at three dominant cleavage sites. Related to Figure 7.

N-terminally, GST-tagged LLO was treated with GzmB for 20 minutes before analysis by SDS-PAGE and Coomassie staining and by αGST immunoblots. Dominant cleavage products were analysed by MassSpectometry and by N-terminal sequencing.

Transparent Methods

Experimental Model Details

In vitro generation of human primary cells and cell culture conditions

Human primary cells cells were generated *in vitro* from peripheral blood mononuclear cells (PBMC) isolated from buffy coats of healthy blood donors (Blood bank, Bern, Switzerland) by Ficoll-Paque™ PLUS (GE Healthcare) density centrifugation. PBMCs were cultured in H5 (RPMI supplemented with 5% heat inactivated human serum (Blood bank, Bern, Switzerland)) and 1% antibiotic/antimycotic solution (Thermo Fisher) for 1 hours at 37°C to allow adhesion of the monocytes. After the incubation, the non-adherent suspension cells were collected and further cultured in H5 supplemented with 5 μM zoledronate (Sigma) + 200 U/ml recombinant human IL-2 (Miltenyi) for 14 days according to (Kondo et al., 2011) to obtain γδ T cells. The cells were diluted and supplemented with fresh IL-2 every 2-3 days. For phenotyping after the expansion, cells were stained with fluorescently conjugated anti γδ–TCR (BD) and assessed by flow cytometry (BD Accuri C6). The frequency of the γδ T cells increased routinely to over 70% during the expansion (data not shown). Lymphokine-activated killer (LAK) cells were generated by culture in H5 supplemented with 200 U/ml recombinant human IL-2 for 14 days according to (Groscurth et al., 1990).

The adherent PBMCs were cultured in H5 for at least 7 days to obtain macrophages (Aurore et al., 2018).

HeLa cells were cultured in DMEM and YT Indy cells in RPMI, both supplemented with 10% FBS (Sigma) and 1% antibiotic/antimycotic solution (Thermo Fisher). For the culture of the shYT Indy line 2 μg/ml puromycin (Sigma) was added (Chiusolo et al., 2017).

Bacterial Strains

Listeria monocytogenes 10403S, *Salmonella Typhimurium* SL1344 and *Mycobacteria bovis* BCG used for infections were grown to mid-log in appropriate medium (Brain Heart Infusion + 50 µg/ml streptomycin for *Listeria*; Luria broth + 50 µg/ml streptomycin for *Salmonella;* Middlebrook 7H9 Broth supplemented with OADC und Tween 80 for *Mycobacteria*). 50 µg/ml Kanamycin was added to grow mutant Listeria (pIMK2 transfected).

Methods Details

Study design

The objective of this study was to identify GNLY independent mechanisms of Gzm antibacterial activity. Without cytosolic delivery by GNLY, the Gzms can only interact with a limited fraction of the bacterial proteins. Therefore, a panel of well-established secreted or externally exposed bacterial virulence mediators were tested for their susceptibility towards GzmB. Bacterial virulent growth after treatment with purified GzmB or Gzm secreting killer cells was tested in primary human macrophages as well as in HeLa cells. A GzmB uncleavable virulent factor was generated to causatively link a Gzm cleavage event with bacterial virulence.

GzmB and PFN Purification

Native human GzmB and perforin was purified from the human NK cell line YT-Indy as described (Lopez Leon et al., 2017). In brief, YT Indy cells were grown in complete medium to about 3×10^9 cells and harvested by centrifugation. The cellular pellet was resuspended in ice-cold relaxation buffer (10 mM PIPES, 0.1 M KCl, 3.5 mM $MgCl^2$, 1 mM ATP, 1.25 mM EGTA, 0.05% BSA, pH 6.8) and disrupted in a nitrogen cavitation bomb at 35 bar at 4°C for 15 minutes. Unbroken cells and nuclei were removed by centrifugation (400 × g for 7 minutes at 4°C) and the post-nuclear

supernatant was spun (15000 × g for 15 minutes at 4° C) to yield the granule pellet. The granule proteins were extracted by repeated freeze/thaw in extraction buffer (1 M NaCl in 20 mM Na-acetate, pH 4.5 containing 2 mM EGTA; 1 ml per $3x10^9$ cells) and the resulting lysate was centrifuged (15000 × g for 10 minutes at 4°C) and filtered (0.45-µm syringe filter, Millipore).

For GzmB purification, the granule extract was diluted (1:20) in S column buffer (50 mM bisTris, 50 mM NaCl, pH 5.8, filtered), load on a S column (HiTrap SP HP; GE) and eluted using a linear NaCl gradient (to 1M). GzmB activity was determined colormetrically using N-Ac-IEPD-pNA (Sigma) in a microplate reader (Synergy H1, Biotek).

For PFN purification, the granule extract was buffer exchanged to PFN-IMAC buffer (1 M NaCl, 20 mM HEPES, 10% betaine, pH 7.4, filtered) using an Econo-Pac 10 DG column (BioRad), then load on a cobalt-IMAC column (HiTrap IMAC HP 5 ml; GE) and eluted on a linear imidazole gradient (to 500 mM). The hemolytic activity of PFN was determined in assay buffer (10 mM Hepes, 0.15 M NaCl, and 0.1% BSA, pH 7.5) incubated with an equal volume of 0.2% human red blood cells in assay buffer containing 5 mM $CaCl_2$ at 37°C for 15 min. The hemoglobin released into the supernatant was detected in a microplate reader (Synergy H1, Biotek). See also hemolysis assays below.

Hemolysis assays

In u-bottomed microtiter plates, recombinant GST-LLO (50 µM) or secreted proteins in the *Listeria* culture supernatant were treated with indicated concentration of GzmB or same volume of PBS as control for 20 minutes before 3,4-dichloroisocoumarin (DCI; Sigma) to a final concentration of 50 µM was added and the samples were incubated on ice for 30 minutes. Human red blood cells (RBCs, Swiss Blood Bank, Bern Switzerland) were washed 3 times and diluted to

a hematocrit of 0.2% in acidic hemolysis buffer (100 mM NaCl, 40 mM $NaPO_4$, 0.5 mg/ml BSA, pH=5.5). An equal volume of diluted RBCs was added to the hemolysins in the microtiter plates and incubated at 37° C for 15 minutes. After the incubation, the plate was spun (500 x g, 3 minutes) and the supernatant was transferred to a flat-bottomed microtiter plate. Hemolysis was assessed by absorbance readings at 405 nm in a plate reader (Synergy H1, Biotek). Specific hemolysis was normalized to positive control lysis induced by 0.1% Triton X-100, corrected by the spontaneous hemoglobin release in buffer only conditions.

Bacterial infections, colony forming unit (CFU) assays, microscopy and TNF-α ELISA

Before infections, bacteria were washed with PBS, resuspended in infection medium (RPMI + 1% BSA + appropriate antibiotics as above) and the OD at 600 nm wavelength was measured to estimate cell density (OD_{600} = 0.1 corresponds to ~$2x10^7$ bacteria /ml).

Macrophages and HeLa cells were infected with *Listeria monocytogenes* 10403S, *Salmonella Typhimurium* SL1344 and *Mycobacteria bovis* BCG (only macrophages) at a multiplicity of infection (MOI) of 10 in infection medium in triplicates. Infections were performed for 30 minutes (*Listeria* and *Salmonella*) or 3.5 hours (*Mycobacteria*). Bacteria were pre-mixed with 0.4 μM GzmB (or equal concentrations of trypsin as control) or killer cells (YT Indy, γδ T and LAK cells) at a killer cell-bacteria ratio of 10 or left untreated for 15 minutes before being added at equal volume to the host cells to perform the infections. In some experiments, the YT Indy and γδ T cells were pretreated for 30 minutes on ice with 25 μM 3,4-dichloroisocoumarin (DCI, Sigma) or DMSO as control. Other co-incubation experiments were performed in presence of 25 μM Ac-IEPD-CHO (Sigma) or by using the granzyme silenced shYT Indy line.

The infected cells were washed with PBS and incubated with gentamicin (25 μg/ml in infection medium) to kill off extracellular bacteria for another 30 minutes (time point 1 hour for *Listeria* and *Salmonella*; 4 hours for *Mycobacteria*). At indicated times, samples were washed with PBS and then hypotonically lysed by adding ice-cold sterile water for 15 minutes. Lysates were serially diluted and spread on LB-Agar plates (Middlebrook-OADC- Agar plates for *Mycobacteria*). After 16 hours incubation at 37° C (2-3 weeks for *Mycobacteria*), CFU were counted. Host cell-free control bacteria samples in infection medium were used to assess extracellular growth.

For microscopy, Listeria were stained with 2 μM CFSE (Sigma) for 30 minutes on ice or with the PKH26 Red Fluorescent Cell Linker kit (Sigma) for 5 minutes at room temperature, washed three times with PBS and resuspended in infections medium. Macrophages and HeLa cells were seeded at a density of 10^5 cells in 500 μl on glass coverslips in 24-well plates and infected with labeled-*Listeria* in presence of 0.2 μM GzmB or not as above. After a PBS wash, cells were incubated for 4.5 hours in infection medium + gentamicin, and then fixed with 1.5% paraformaldehyde in PBS. Fixed cells were washed twice with PBS and then stained with phalloidin-AF594 (150 nM, ThermoFisher) and DAPI (1 μg/ml, Sigma) for 30 minutes at room temperature in the dark.

To assess cell death, infected cells were fixed in cold methanol (-20° C) for 15 minutes, washed twice with PBS and then stained with the CytoDEATH M30 antibody (Roche) and Hoechst (1 μg/ml, Sigma) for 1 hour at room temperature in the dark. After the primary antibody, cells were washed with PBS and then counterstained with anti-mouse IgG-AF594 (R&D Systems) for 30 minutes at room temperature.

To assess co-endocytosis, HeLa cells were infected with red-labeled *Lm* in presence of GzmB-AF488 as above. After PFA fixation, cells were stained with Hoechst (1 μg/ml, Sigma) for 30 minutes at room temperature in the dark.

All stained cover slips were washed twice with PBS before mounting in vectashield (vectorlabs) and analysis by confocal microscopy (Leica SP5).

TNF-α levels were measured by subjecting serial dilutions of BCG infected macrophage supernatants to sandwich ELISA (R&D Dual kit) according to the manufacturer's recommendations

Assessment of GzmB secretion by killer cells in response to bacterial exposure by immunoblot and ELISA

Killer cells (YT Indy, activated γδ T cells, LAK cells) were incubated for 60 minutes at 37° C with Lm or *Salmonella* SL1344 at a bacteria-lymphocyte ratio of 1:10 and then centrifuged (400 x g, 10 minutes) to separate the cellular pellet from the supernatant that was additionally filtered (0.45 μm). The pellet was lysed in RIPA buffer. Supernatant and pellet were assessed by immunoblots using mouse anti-GzmB (clone B18.1; Enzo Life Sciences) and mouse anti-actin antibodies (Thermofisher), respectively, (in **Figures S5** and **S6**); or using rat anti-GzmB (clone 496B, Invitrogen) and rabbit anti-GAPDH (Sigma), in **Figures 4** and **5**. All secondary HRP-conjugated antibodies were purchased from R&D Systems. Blots were analyzed using the Syngene G:Box F3 imager.

GzmB levels were measured by subjecting serial dilutions of activated killer cell supernatants to sandwich ELISA (R&D Dual kit) according to the manufacturer's recommendations

Assessing host cell viability in response to SL1344 infection by BCECF fluorescence release assays

Fluorescence release assays using BCECF-AM (Sigma) were performed according to (Aurore et al., 2018). Briefly, human macrophages, seeded in flat-bottom 96-well microplates, were labelled before the infection with 2,7-Bis(2-carboxyethyl)-5(6)-carboxyfluorescein acetoxymethyl ester (BCECF-AM; Sigma) for 30 minutes in RPMI at 37° C (Kolber et al., 1988). After three washes with PBS, the cells were infected with SL1344 as above. After removal of extracellular bacteria by gentamicin treatment, the released fluorescence at indicated time points was measured in the supernatant by plate reader at the 488-Exitation/530-Emission setting.

Assessment of bacterial substrate cleavage, production of recombinant LLO and priming of *Listeria* supernatants

E. coli Bl21 harboring GST-fusion protein in pGEX4Ti were induced for 60 minutes at 37°C with 0.25 mM isopropyl β-D-1-thiogalactopyranoside (IPTG) in LB containing 100 μg ampicillin and 2% glucose, then were washed, resuspended in 20 mM NaCl, 10 mM Tris, pH 7.4 supplement with 0.1 mg/ml lysozyme (Sigma) and incubated for 30 min on ice. After three freeze and thaw cycles, the crude lysates were treated with indicated concentration of GzmB for 30 min or with trypsin (from bovine pancreas, Sigma) for 5 minutes on 37° C. Reactions were stopped by boiling in SDS-PAGE loading buffer. Samples were analyzed by immunoblot using mouse anti-GST antibody (BD). For the assessment of LLO hemolytic activity and the identification of GzmB cleavage sites, GST-tagged LLO was purified using a GSTrap HP column (GE Healthcare) following the manufacturer`s recommendations.

To assess the degradation of native secreted proteins, *Listeria* cultures were grown to the mid-log phase, washed 3 times with PBS and resuspended in RPMI. *Listeria* in RPMI were incubated in a

shaking incubator at 37° C for 1 to 4 hours before the supernatant was cleared by centrifugation (1000 x g for 5 minutes) and filtration (0.45 μm). Cleared supernatants were treated with GzmB as above and analyzed by immunoblot using mouse anti-Iap (p60, antibodies-online.com) and rabbit anti-LLO antibodies (Abcam). Secondary HRP-conjugated antibodies were from R&D Systems. Blots were analyzed using the Syngene G:Box F3 imager.

Generation of a GzmB-uncleavable LLO mutant and expression in a LLO-deficient *Listeria* strain

GST-tagged LLO was treated with 0.2 μM GzmB for 20 minutes before the cleavage sites were visualized by SDS-PAGE and Coomassie staining and anti-GST immunoblots. The N-terminal cleavage sites were analyzed by Mass Spec (Proteomics core facility, University of Fribourg, Switzerland). The C-terminal fragments were subjected to N-terminal sequencing (PROTEIN ANALYTICS, University of Giessen, Germany). The three dominant cleavage site aspartic acids were mutated to alanines by two-step overlap PCR. Full-length (including the natural ribosome binding site) wild-type and mutated LLO was cloned into pIMK2 (Monk et al., 2008) at the BamH1/Xho1 sites and then transfected into LLO-deficient *Listeria.*

Statistics

All experiments were performed in triplicates and were at least once independently repeated. Data are presented as means ± SEM. Comparisons between the different groups were performed with two-tailed unpaired Student`s t tests (using Microsoft Excel). P values are indicated in the Figures. *P* values of less than 0.05 were considered significant.

References

Aurore, V., Caldana, F., Blanchard, M., Kharoubi Hess, S., Lannes, N., Mantel, P.Y., Filgueira, L., and Walch, M. (2018). Silver-nanoparticles increase bactericidal activity and radical oxygen responses against bacterial pathogens in human osteoclasts. Nanomedicine *14*, 601-607.

Chiusolo, V., Jacquemin, G., Yonca Bassoy, E., Vinet, L., Liguori, L., Walch, M., Kozjak-Pavlovic, V., and Martinvalet, D. (2017). Granzyme B enters the mitochondria in a Sam50-, Tim22- and mtHsp70-dependent manner to induce apoptosis. Cell death and differentiation *24*, 747-758.

Groscurth, P., Diener, S., Stahel, R., Jost, L., Kagi, D., and Hengartner, H. (1990). Morphologic analysis of human lymphokine-activated killer (LAK) cells. Int J Cancer *45*, 694-704.

Kolber, M.A., Quinones, R.R., Gress, R.E., and Henkart, P.A. (1988). Measurement of cytotoxicity by target cell release and retention of the fluorescent dye bis-carboxyethyl-carboxyfluorescein (BCECF). Journal of immunological methods *108*, 255-264.

Kondo, M., Izumi, T., Fujieda, N., Kondo, A., Morishita, T., Matsushita, H., and Kakimi, K. (2011). Expansion of human peripheral blood gammadelta T cells using zoledronate. Journal of visualized experiments : JoVE.

Lopez Leon, D., Fellay, I., Mantel, P.Y., and Walch, M. (2017). Killing Bacteria with Cytotoxic Effector Proteins of Human Killer Immune Cells: Granzymes, Granulysin, and Perforin. Methods in molecular biology *1535*, 275-284.

Monk, I.R., Gahan, C.G., and Hill, C. (2008). Tools for functional postgenomic analysis of listeria monocytogenes. Applied and environmental microbiology *74*, 3921-3934.
